# An improvement of real-time polymerase chain reaction system based on probe modification is required for accurate detection of African swine fever virus in clinical samples in Vietnam

**DOI:** 10.5713/ajas.19.0525

**Published:** 2019-12-24

**Authors:** Ha Thi Thanh Tran, Anh Kieu Dang, Duc Viet Ly, Hao Thi Vu, Tuan Van Hoang, Chinh Thi Nguyen, Nhu Thi Chu, Vinh The Nguyen, Huyen Thi Nguyen, Anh Duc Truong, Ngoc Thi Pham, Hoang Vu Dang

**Affiliations:** 1 Department of Biochemistry and Immunology, National Institute of Veterinary Research (NIVR), 86 Truong Chinh, Dong Da, Hanoi 100000, Vietnam

**Keywords:** African Swine Fever, Real-time Polymerase Chain Reaction, Conventional Polymerase Chain Reaction, PAMs Cell, Virus Isolation, Molecular Diagnosis

## Abstract

**Objective:**

The rapid and reliable detection of the African swine fever virus (ASFV) plays an important role in emergency control and preventive measures of ASF. Some methods have been recommended by FAO/OIE to detect ASFV in clinical samples, including real-time polymerase chain reaction (PCR). However, mismatches in primer and probe binding regions may cause a false-negative result. Here, a slight modification in probe sequence has been conducted to improve the qualification of real-time PCR based on World Organization for Animal Health (OIE) protocol for accurate detection of ASFV in field samples in Vietnam.

**Methods:**

Seven positive confirmed samples (four samples have no mismatch, and three samples contained one mutation in probe binding sites) were used to establish novel real-time PCR with slightly modified probe (Y = C or T) in comparison with original probe recommended by OIE.

**Results:**

Both real-time PCRs using the OIE-recommended probe and novel modified probe can detect ASFV in clinical samples without mismatch in probe binding site. A high correlation of cycle quantification (Cq) values was observed in which Cq values obtained from both probes arranged from 22 to 25, suggesting that modified probe sequence does not impede the qualification of real-time PCR to detect ASFV in clinical samples. However, the samples with one mutation in probe binding sites were ASFV negative with OIE recommended probe but positive with our modified probe (Cq value ranked between 33.12–35.78).

**Conclusion:**

We demonstrated for the first time that a mismatch in probe binding regions caused a false negative result by OIE recommended real-time PCR, and a slightly modified probe is required to enhance the sensitivity and obtain an ASF accurate diagnosis in field samples in Vietnam.

## INTRODUCTION

African swine fever (ASF) is a pernicious and devastating disease of the world swine industry. It has been demonstrated that this disease is caused by an enveloped DNA virus, and this virus replicates in the cytoplasm of infected cells in pigs [1,2]. It is not only a recognized DNA arbovirus, but also the member of the family *Asfarviridae* and genus *Asfivirus* [2–4]. Many previous studies have reported that this virus infects both domestic pigs and wild boars, and it can easily be transmitted by many pathways such as mosquitoes, ticks, or other arthropods.

Additionally, as a highly contagious virus, pigs exposure to African swine fever virus (ASFV) resulted in up to 100% of morbidity in pigs, and the mortality of ASF depended on the virulence of the virus, the host, and transmission cycles [4,5]. The first case of ASFV was reported in Kenya in 1921 [1], and then, it was confined in Africa until it has rapidly spread to Eurasia in the middle of the last century, and South America and the Caribbean in the late of the last century [1]. To date, 24 genotypes of ASFV have been identified [1,3,5,6]. From early-February 2019, an ASF outbreak in Vietnam was reported officially, and to date, the disease was reported in 63/63 provinces/cities of Vietnam. Over 3.7 million pigs have been culled in infected farms, suggesting the risk of the spread of this virus. Many active measures were conducted by Vietnamese Government to inhibit the rapid spread of ASFV, and the improvement of ASFV diagnostic methods is one of the essential steps to control and prevent the ASFV in Vietnam.

Until now, a vaccine or treatment is not yet available for ASFV. Therefore, rapid and reliable diagnosis plays a vital role in control measures to reduce the spread of ASFV [1,7]. According to World Organization for Animal Health (OIE), laboratory techniques of ASFV diagnosis were divided into two groups i) virological tests, including virus isolation combined with haemadsorption (HAD) test and viral genome detection, i.e., conventional polymerase chain reaction (PCR), real-time PCR, enzyme-linked immunosorbent assay (ELISA) and ii) serological tests such as ELISA, immunoblotting test [2] and indirect fluorescent antibody (IFA) [7,8]. The highest specific diagnosis of ASFV is HAD test. It is recommended that virus isolation combined with HAD test is powerful tool to verify the positive results of conventional PCR, real-time PCR, ELISA, and immunofluorescence test (IFAT).

Additionally, the HAD test is “gold standard” method and a positive HAD test is definitive for ASFV diagnosis. Although, virus isolation in combination with HAD test using porcine alveolar macrophages (PAMs) cells is a prestigious method for ASFV diagnosis, the performance of this test requires excellent laboratory skills and takes more than a week to obtain the result [3,7,8]. One of the best sensitive tests for ASFV detection in real-time PCR technique. The real-time PCR assays have been commonly using due to their efficiently, faster, high sensitivity and specificity, and these assays have been adopted for routine diagnosis in national and reference laboratories [1,7,8]. In recent years, an improved real-time PCR assay has shown higher diagnostic sensitivity when compared to original one. Based on a universal probe, this method has demonstrated superior sensitivity when detecting experimental and field samples [1,3,7–9]. According to FAO, while the HAD test is the highest specific test, real-time PCR is considered to be highest sensitive method, indicating that a combination of these genome detective methods is necessary to accurately diagnose ASFV in clinical samples.

However, recent studies on real-time PCR system has shown that mismatches in primer and probe binding regions may directly affect real-time PCR qualification, leading a false negative result. It was indicated that a single mismatch on primer or probe binding site causes false-negative results, and up to 33% were recognized [10]. Mainly, a conflicting result between real-time PCR and the reference method, direct IFAT, was reported [11] in which the mismatches in primer and probe binding region resulted in a quantification error up to 60%. These studies have shown a weak point of this method on virus detection in the field samples. Therefore, it is necessary to update the current real-time PCR assay by modification of the primer and probe binding region to allow detection of the currently circulating ASFV in field samples.

In the current study, a novel probe with slight modification based on our data and some references have been tested in comparison with the original OIE recommended probe to bring the best understanding underlying the adverse effects of a mismatch in probe binding site on accurate diagnosis of ASFV in field samples in Vietnam.

## MATERIALS AND METHODS

### Animal care

The study was conducted in compliance with the institutional rules for the care and use of laboratory animals and using a protocol approved by the Ministry of Agriculture and Rural Development (MARD) Vietnam (TCVN 8402:2010).

### Cell and virus isolation

Primary PAMs were collected from the lung of pigs cultured in RPMI 1640 medium (Thermo Scientific, Waltham, MA, USA) supplemented with 10% fetal cattle serum, at 37°C with 5% CO_2_ and adherent cell populations were selected after overnight culture according to the OIE guidelines.

Seven ASFV positive spleen samples checked by conventional PCR using PPA1/PPA2 primer sets were further examined by virus isolation in combination with haemadsorption (HAD) assay, and subsequently confirmed by conventional PCR and sequencing analysis were used for this study. Briefly, these cells were obtained and resuspended in Dulbecco’s modified Eagle’s medium (DMEM, Thermo Fisher Scientific, Waltham, MA, USA) supplemented with streptomycin (Sigma-Aldrich, Louis, MO, USA), ampicillin (Sigma-Aldrich, USA) and 5% fetal calf serum (Sigma-Aldrich, USA) to a final concentration of 1×106 cells/mL. Six-well culture plates (Thermo Scientific, USA), containing 2 mL of the cell suspension, were inoculated with 50 μL of a clarified 10% spleen homogenate. After three days of incubation at 37°C, in an atmosphere with 5% CO_2_, the first passage virus was harvested by freezing and thawing. For the second passage, 50 μL of this virus harvest was added to 2 mL fresh PAMs, then incubated and harvested as described for the first passage. For the third passage, 50 μL of this virus harvest were added to 2 mL fresh PAMs, then incubated at 37°C, in an atmosphere with 5% CO_2_. After one-day incubation, a 20-μL preparation of 1% homologous red blood cells (RBC) in buffered saline was added to each well. The plates were examined for HAD and cytopathic effects over four days in PAM cells according to the recommendation by OIE [7].

### Conventional polymerase chain reaction and sequencing analysis

ASFV genomic DNA was extracted by using the QIAamp DNA Mini Kit (QIAgen, Hilden, Germany) from cell supernatants or spleen homogenate. Conventional PCR was performed with specific primers, PPA1/PPA2, and King’s primers as shown in Table 1 according to the recommendation by OIE. Briefly, ASFV DNA was amplified by PCR using the ASF diagnostic primers PPA1 and PPA2 and King’s primers. PCR was carried out in Agilent PCR System (Agilent, Santa Clara, CA, USA) using Taq polymerase (Thermo Scientific, USA), according to the manufacturer’s instructions. Thermal conditions for performing PCR are as follows: an initial incubation at 95°C for 10 min; 40 cycles of denaturation at 95°C for 15 s, annealing at 58°C (King’s primers) and 62°C (PPA1/PPA2 primers) for 30s, and extension at 72°C for 30 s; and final incubation 72°C for 7 min. Amplification products were electrophoresed on a 1.5% agarose gel against a 100 bp DNA ladder marker (Thermo Scientific, USA) and visualized by UV irradiation and ethidium bromide staining (Sigma-Aldrich, USA). Amplicons of the correct size were excised from the agarose gel and purified using the QIAQuick gel extraction kit (QIAgen, Valencia, CA, USA) according to the manufacturer’s specifications for sequencing. The chromatograms of amplicons and probe binding site sequences were analyzed using BioEdit and DNAstar program. The nucleotide identity of the probe binding site sequence of the ASFV strain in Vietnam in comparison with other sequences were performed using Blast tool at the National Center for Biotechnology Information (NCBI) database and using the information of published sequence, including China/Jilin/2018/boar (GenBank accession no. MK189456), CN201801 (GenBank accession no. MH722357), Russia/Irkutsk 2017 (GenBank accession no. KY963545), Congo/Ndjassi-77 (GenBank accession no. KM236553), Nigeria/Imo/1006/2017 (GenBank accession no. MG209617), BOT/1/99 (GenBank accession no. AF504886), Uganda/Ug64 (GenBank accession no. FJ174383), Kenya/Hinde II (GenBank accession no. AF449480), Kenya/Killean/1 (GenBank accession no. AY351550), and Kenya/Trench (GenBank accession no. AY351547).

### Real-time polymerase chain reaction using two different probes

The real-time PCR was carried out on an Agilent AriaMx Real-Time PCR System (Agilent, USA) according to the OIE-recommended procedure described in King et al [12] using two different probes, one was the original OIE probe, and other a novel modified probe. The primer- and probe-sequences are shown in Table 1. Positive amplification control consisted of known DNA of ASFV positive, and negative amplification control included of nuclease-free sterile water.

## RESULTS

### Confirmation of seven positive samples by virus isolation in combined with HAD test

**Figure 1:**
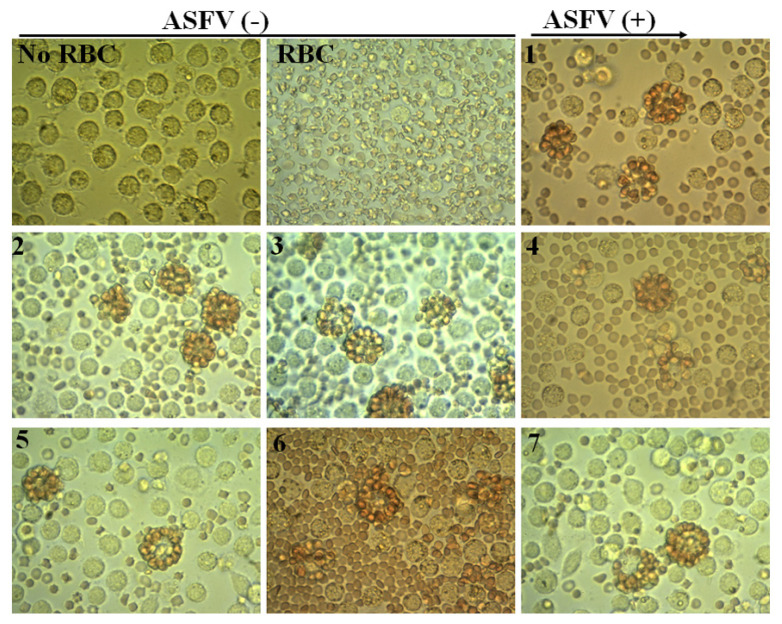
Virus isolation is combined with the HAD test. No RBC, Mock-non infected cells without red blood cell at 72 h; RBC, Mock-non infected cells with red blood cell observed at 72 h; 1–7, hemadsorption in the culture of PAM cells infected with seven ASFV positive samples (Original magnification, 400×). HAD, haemadsorption; RBC, red blood cells; PAM, porcine alveolar macrophages; ASFV, African swine fever virus.

**Figure 2:**
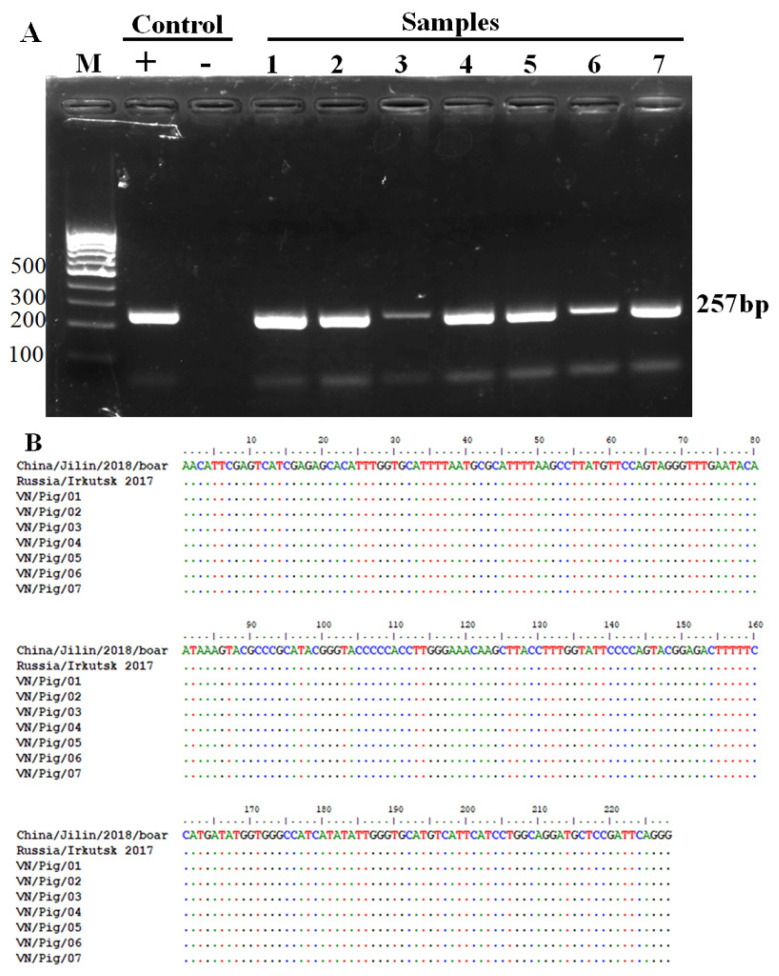
Confirmation of positive HAD by conventional PCR and sequencing analysis (A) The conventional PCR to detect ASFV after 3 passages of virus-infected cell culture using PPA1/PPA2 primers as described by OIE and (B) Multiple sequence alignment of p72 gene amplified by PPA1/PPA2 primers in Vietnam ASFV strains with reference ASFV strains, including China/Jilin/2018/boar (GenBank accession no. MK189456), CN201801 (GenBank accession no. MH722357) and Russia/Irkutsk 2017 (GenBank accession no. KY963545), dot indicate identify with ASFV references sequences. PCR, polymerase chain reaction; ASFV, African swine fever virus.

Seven positive samples checked by conventional PCR (data do not show) were further analyzed by virus isolation combined with HAD test according to OIE protocol. Subsequently, positive HAD was verified by conventional PCR and sequencing analysis using PPA1 and PPA2 primers. The results are shown in Figure 1 and 2. As seen in Figure 1 a strong positive HAD was observed in seven ASF isolates, and the titer for the first passage stock ranged 10^5.5^ to 10^6.5^ HAD_50_/mL. To verify these data, we performed conventional PCR and then sequencing analysis using PPA1 and PPA2 primers to confirm positive HAD as recommended by the OIE. These results are shown in Figure 2. A positive 257-bp product was obtained in all isolates, as seen Figure 2A. Further confirmation using sequencing method are shown in Figure 2B. These results indicated that the nucleotide sequences of seven ASF isolates shared 100% identities with the following strains: China/Jilin/2018/boar isolated in China in 2018 and Irkutsk 2017 from Russia based on the fragment of p72 gene amplified by specific PPA1/PPA2 primer (Figure 2B). These results indicated that seven positive confirmed samples were suitable for establishment of novel real-time PCR to detect ASFV in clinical samples.

### Identification of a mutation in probe binding sites of ASFV isolated in Vietnam

**Figure 3:**
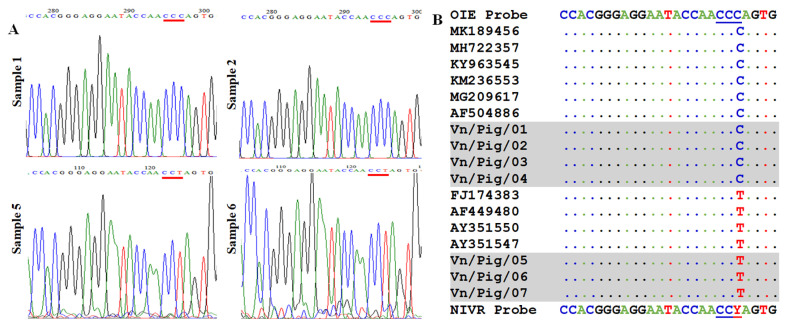
Identification of mismatch in probe binding regions (A) The sequencing results of ASFV strains in Vietnam after amplification with a specific set of primers described by King et al [12]. (B) Multiple sequence alignment of the probe binding site of Vietnam ASFV strains with reference ASFV strains from the NCBI. ASFV, African swine fever virus; NCBI, National Center for Biotechnology Information.

To recognize the mutation in probe binding site, seven positive confirmed samples were further checked by sequencing analysis using King’s primer set. The region sequences for probe binding are shown in Figure 3. Among seven positive confirmed samples, we found that four samples have no mismatch and three samples contained one mutation in probe binding site as shown in Figure 3 in which peak of “C” was noted in samples 1 and 2 when a strong peak “T” was observed in sample 5 and 6. Also, the alignment of seven samples with some references can be seen in Figure 3B, suggesting that a mismatch in probe binding sites may cause false-negative results by real-time PCR if using original OIE probe. A slight modification in probe sequence is required to detect all seven positive confirmed samples with or without mutation by real-time PCR.

### Improvement of real-time polymerase chain reaction qualification by novel modified Taq-man probe

A new degenerate probe based on our data and some references was designed as seen in Table 1 in which a single mismatch in probe binding sequence was replaced by a “Y” (Y = C or T) to detect ASFV with or without the mutation. A real-time PCR using two different probes, original OIE, and modified probes, was performed based on the OIE manual for ASF diagnostics (Table 2). The results have shown that 4/7 positive confirmed samples were ASFV positive with both probes with similarly cycle quantification (Cq) value (OIE probe: sample 1 to 4 Cq value ranked 22.76 to 25.82, and modified probe Cq value of sample 1 to 4 ranked 22.18 to 25.08; Table 2). A good correlation of Cq values between two probes was observed in this study, indicating that replacement of “Y” in probe sequence does not impede the qualification of real-time PCR to detect ASFV in clinical samples. However, 3/7 samples were ASFV negative with OIE probe but positive with our modified probe (Cq value ranked between 33.12–35.78, Table 2). These findings suggested that a mismatch in probe binding site resulted in false-negative diagnosis by OIE recommended probe, and a slight modification is required to enhance the sensitivity of real-time PCR for accurate diagnosis of ASFV in clinical samples in Vietnam.

## DISCUSSION

ASF is a highly contagious viral disease of swine, and the mortality and morbidity were up to 100% in domestic pigs [13]. ASF is a very complex and lethal viral disease for which no vaccine is available to prevent the infection. From early-February 2019, an ASF outbreak in Vietnam was reported officially, and currently, this disease continues to spread quickly to 58/63 provinces and cities of Vietnam. Over 2.5 million pigs have been culled from infected farms, suggesting the risk of the spread of this virus. Many control measures were conducted by Vietnamese Government to inhibit the potential to spread rapidly to new, uninfected areas. ASF is causing significant damage to the pig industry and trade restrictions, and the improvement of ASF diagnostic strategy is one of the essential steps to control and prevent the ASF in Vietnam. Control measures emphasize the need for early, rapid, high rates of sensitive and specific diagnosis in ASFV.

Real-time PCR and conventional PCR assay are the most widely used assay and also recommended by OIE for virological and molecular diagnosis of ASF [7,8]. Both PCRs are useful and generally more applicable, especially in less equipped laboratories and are valuable tools for routine diagnosis of the disease [1]. However, recent studies have indicated that the OIE-recommended conventional PCR to detect ASFV had low sensitivity, most likely due to an imperfect match of the primers with the target sequences of some ASFV genotypes [1,14], increasing the contamination risk, and is being broadly replaced by the real-time PCR system. Although OIE real-time PCR has shown excellent sensitivity and specificity rates, the high fidelity of the method is slightly decreased when the samples showed low or weak ASFV-positive [12,15,16]. It may be related to epidemiological or virulent ASFV such as chronically infected pigs, low virulence of ASFV in infected pigs or low level of ASFV in the infected pig [3,9]. In this study, we improved the sensitivity of real-time PCR detection by slight modification based on OIE recommended probe for the diagnosis of circulating ASFV strains in Vietnam. These results showed that four out of seven positive confirmed samples were ASFV positive with both probes with similarly Cq value (OIE probe: Cq value of sample 1 to 4 ranked 22.76 to 25.82 and novel modified probe: Cq value of sample 1 to 4 ranked 22.18 to 25.08; Table 2). A good correlation of Cq values between two probes was observed in this study, indicating that replacement of “Y” in probe sequence does not impede the qualification of real-time PCR to detect ASFV in clinical samples. Multiple sequence alignment of probe binding sites showed that the OIE-recommended probe is not all conserved to cover these genotype II ASFV strains. BLAST searches of the modified ASFV probe confirmed that they target highly conserved regions of the ASFV vp72 gene sequences in Vietnam and present in the current NCBI nucleotide sequence collection. Our further results demonstrated that three samples were ASFV positive with HAD, conventional PCR, sequencing analysis, and real-time PCR using OIE modified probe (33.12, 34.15, and 35.78) but a negative result was obtained by OIE-recommended real-time PCR.

Additionally, it indicated that the lower sensitivity of the OIE-recommended probe or virulent of ASFV might be due to the presence of a nucleotide mismatch of the probe with the target sequences of some ASFV isolates, which is consistent with what we presented in our sequence alignment. As shown in Figure 3B, the OIE recommended probe display the same mismatch in probe binding sites with several previous ASFV isolates in NCBI database, including Kenya 2001/II (genotype II) (GenBank accession no. AF449480) and Uganda 1964 (genotype II) (FJ174383). Moreover, previous reports have demonstrated that a slight modification (Y = C/T) in probe binding regions for detection of human Norovirus GII or Rhinoviruses do not interfere the qualification of real-time PCR and significant increasing in the sensitivity of detection were recognized [1,14], suggesting that an improvement of real-time PCR system by a probe modification is essential to enhance the sensitivity of ASFV detection in clinical samples in Vietnam.

## CONCLUSION

In summary, we improved successfully the real-time PCR system by probe modification to enable the detection of the circulating ASFV strains in Vietnam. The novel modified probe based on our current data and some references in GenBank has shown higher sensitivity than the original one, recognizing clinical ASFVs with and without a mutation in probe binding regions. Additionally, a combination of the real-time PCR with the OIE modified probe and the virus isolation in PAMs cell with HAD test is powerful tool for rapid and reliable diagnosis as well as genomic surveillance in Vietnam.

## Figures and Tables

**Table 1:** Primer and probe sequence used in this study

Name	Sequence (5′–3′)	Note
PPA1	AGT TAT GGG AAA CCC GAC CC	[7]
PPA2	CCC CTG AAT CGG AGC ATC CT	
King’s primer F	CTG CTC ATG GTA TCA ATC TTA TCG A	
King’s primer R	GAT ACC ACA AGA TC(AG) GCC GT	
OIE probe	FAM-CCA CGG GAG GAA TAC CAA **CCC** AGT G-TAMRA	
NIVR probe	FAM-CCA CGG GAG GAA TAC CAA **CCY** AGT G-TAMRA	This study

OIE, World Organization for Animal Health; NIVR, National Institute of Veterinary Research, Viet Nam.

**Table 2:** Comparison of cycle quantification values between real-time polymerase chain reaction using original OIE probe and a real-time polymerase chain reaction using a slightly modified probe for African swine fever virus detection in clinical samples

Samples	Cq values	

	OIE probe	NIVR Probe
Positive control	25.80	25.78
Negative control	No Cq	No Cq
Sample 1	22.76	22.18
Sample 2	23.65	23.33
Sample 3	24.40	24.07
Sample 4	25.82	25.08
Sample 5	No Cq	33.12
Sample 6	No Cq	34.15
Sample 7	No Cq	35.78

Cq, cycle quantification; OIE, World Organization for Animal Health; NIVR, National Institute of Veterinary Research, Viet Nam.
